# COVID-19 Exposure Unmasking Systemic Amyloidosis With Hepatic Predominance

**DOI:** 10.7759/cureus.31092

**Published:** 2022-11-04

**Authors:** Jose R Russe-Russe, Chiya Abramowitz, James R Pellegrini, Alejandro Alvarez Betancourt, Ricky Cohen, Michael Baldino, Ronald Crandall, Deepthi Kagolanu, Jose Mejia, Kaleem Rizvon

**Affiliations:** 1 Internal Medicine, Nassau University Medical Center, East Meadow, USA; 2 Internal Medicine, New York Institute of Technology College of Osteopathic Medicine (NYITCOM), Glen Head, USA; 3 Internal Medicine, Nassau University Medical Center, Glen Head, USA; 4 Gastroenterology, Nassau University Medical Center, East Meadow, USA

**Keywords:** hepatology, amyloid plaques, liver function, covid-19, gastrointestinal amyloid, clinical infectious medicine

## Abstract

Amyloidosis is characterized by depositing insoluble fibrillar proteins that misfold into beta-pleated sheets. This phenomenon occurs on a systemic or local level and may interfere with the function of various organs, including the heart, kidneys, and liver. Among those presenting with amyloidosis, hepatic, gastrointestinal, renal, cardiac, vitreous, and immunological involvement may occur. These manifestations are linked to several clinical presentations, varying from abdominal pain and hepatomegaly to restrictive cardiomyopathy and chronic renal failure. The two most common types of amyloid proteins are amyloid light chain (AL) and serum amyloid A (AA) proteins. AL produced by immunoglobulin light chains kappa and lambda (κ, λ) circulate systemically and accumulate in organs. At the same time, serum AA proteins are acute-phase reactants seen in infectious, chronic inflammatory states. In an immune-mediated infection such as COVID-19, serum AA levels may be a predictive factor of disease severity and a valuable biomarker to monitor the clinical course of COVID-19 patients. This report highlights a case in which infection with COVID-19 provoked an effective immune response that may have contributed to the accelerated progression of systemic amyloidosis with hepatic involvement. The study further investigates the involvement of AL and AA proteins in COVID-19 infections, including their role in synergistically exacerbating an already grueling clinical course.

## Introduction

Amyloidosis is characterized by the deposition of insoluble fibrillar proteins that misfold into beta-pleated sheets instead of their correct alpha-helical configuration; this may occur systemically or locally [1]. When viewed under polarized light with congo red stained tissue, these linear, non-branching filaments of beta-pleated sheets have apple-green birefringence. [2]. When the insoluble filaments accumulate and deposit in the interstitial space, they damage tissues and interfere with their function [2].

There are about 60 types of amyloid proteins. However, the most common types are amyloid light chain (AL) and serum amyloid A (AA) protein [2]. AL is derived from monoclonal plasma cells in the bone marrow and produced by immunoglobulin light chains composed of kappa or lambda (κ, λ) proteins, also known as primary systemic amyloidosis [1]. AL deposits in many organs, but the main affected organ usually denominates the clinical picture [2]. AA are acute phase reactants (APRs), which play an integral role in the immune response to inflammation, also known as secondary systemic amyloidosis [3]. AA proteins derived from serum amyloid-associated proteins are APRs increased in infectious, chronic inflammatory states, and neoplasias [3].

In hepatic amyloidosis, there is accumulation and deposition of amyloid protein, ultimately interfering with normal liver function. The assembly of amyloid fibrils usually starts in the periportal space of Disse [4]. Compression and atrophy of hepatocytes block the sinusoids, producing portal hypertension as systemic amyloidosis progresses within the liver parenchyma, and patients commonly present with hepatomegaly (HM) and a poor prognosis [4]. Other common symptoms can include fatigue, abdominal pain, edema, anorexia, early satiety, and nausea. We present a case of systemic amyloidosis primarily affecting the liver, and we hypothesize that this underlying condition was enhanced by a recent infection with COVID-19.

## Case presentation

A 53-year-old male with a past medical history of type 2 diabetes mellitus, hypertension, hyperlipidemia, schizophrenia, and polysubstance abuse of alcohol, cocaine, and marijuana presented with gradually increasing jaundice, postprandial nausea, dull and intermittent right upper quadrant pain, and weight loss over three months. A month prior, the patient had previously been admitted for postprandial nausea and weight loss of 30 lbs with initial laboratory tests showing antibodies to COVID-19 but had left the hospital against medical advice.

**Figure 1:**
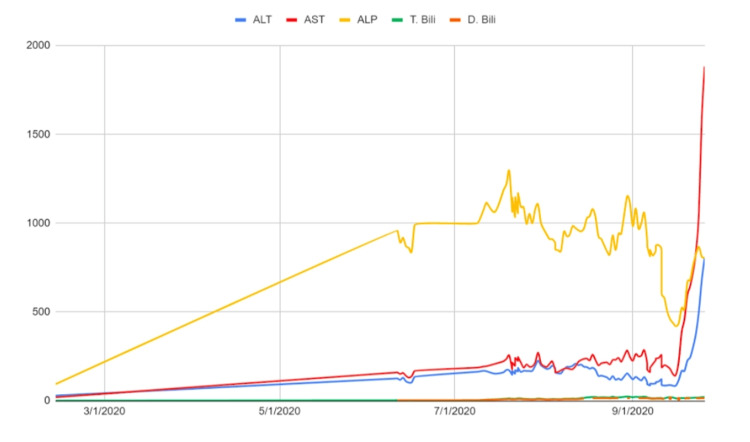
A graph summarizing the liver-related tests during the hospital course of the patient. ALT: Alanine transaminase (Normal 7-40U/L), AST: Aspartate aminotransferase (Normal 13-40U/L), ALP: Alkaline phosphatase (Normal 46-116U/L), T.Bili: Total bilirubin (Normal 0.3-1.2mg/dL), and D.Bili: Direct bilirubin (Normal 0-0.3mg/dL)

On physical examination, the patient was hemodynamically stable with evident scleral icterus, multiple excoriations on bilateral upper and lower extremities, distended abdomen with shifting dullness and fluid wave, and the liver was palpable 3 cm below the costal margin. Biochemical tests on admission revealed the following results: total bilirubin of 3.1mg/dL, direct bilirubin of 2.4mg/dL, alkaline phosphatase of 960U/L, alanine transaminase (ALT) of 126U/L, aspartate aminotransferase (AST) of 160U/L, and ɣ-glutamyl transferase of 2,205U/L (Table 1). These levels trended upward throughout the patient’s hospital stay (Figure 1). These results suggested acute liver injury but were inconclusive for a definitive diagnosis and the patient was initially managed with conservative therapy. The patient tested positive for IgG antibodies against COVID-19; in May 2020, no molecular testing was readily available as standardized testing. The lack of collateral information and in-depth testing early on in the pandemic limited the diagnosis of active COVID-19 infection. Antibodies to human immunodeficiency virus (HIV), cytomegalovirus, herpes simplex virus, hepatitis B, C, and E, and autoimmune markers including antinuclear antibodies, mitochondrial, smooth muscle, tissue transglutaminase, liver, and kidney microsomal antibodies, were all negative (Table 1).

**Table 1:** Laboratory test result

Laboratory Tests	Results	Reference Range
Total bilirubin	3.1 mg/dL	0.3-1.2mg/dL
Direct bilirubin	2.4mg/dL	0-0.3mg/dL
Alkaline phosphatase (ALP)	960U/L	46-116U/L
Alanine transaminase (ALT)	126U/L	7-40U/L
Aspartate aminotransferase (AST)	160U/L	13-40U/L
ɣ-glutamyl transferase (GGT)	2205U/L	12-72U/L
SARS-CoV-2 IgG	Detected	Not detected
Human Immunodeficiency Virus (HIV)	Negative	Negative
Cytomegalovirus (CMV)	Negative	Negative
Herpes Simplex Virus-1 (HSV-1)	Negative	Negative
Herpes Simplex Virus-2 (HSV-2)	Negative	Negative
Hepatitis B	Negative	Negative
Hepatitis C	Negative	Negative
Hepatitis E	Negative	Negative
Antinuclear Antibody (ANA)	Negative	Negative
Anti-Mitochondrial Antibody (AMA)	Negative	Negative
Anti-Smooth Muscle Antibody (ASMA)	Negative	Negative
Anti-Tissue Transglutaminase	Negative	Negative
Anti-Liver Kidney Microsomal	Negative	Negative
Serum Light Chain Analysis:		
Kappa	247.8mg/dL	0.33–1.94 mg/dL
Lambda	16.2mg/dL	0.57–2.63 mg/dL
M Spike on serum protein electrophoresis (SPEP)	Negative	Negative
24-hour urine kappa/lambda ratio	8.64 mg/dL	0.26 – 1.65 mg/dL

**Figure 2:**
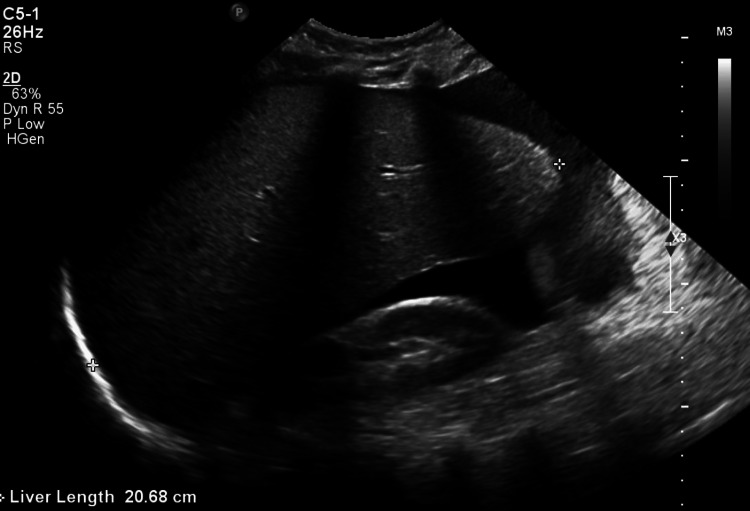
Abdominal ultrasound (US) findings: The visualized portions of the liver are homogeneous. There is hepatomegaly measuring 20.68cm and subtle hepatic surface nodularity suggestive of cirrhosis with a moderate volume of ascites (prior to conservative therapy).

**Figure 3:**
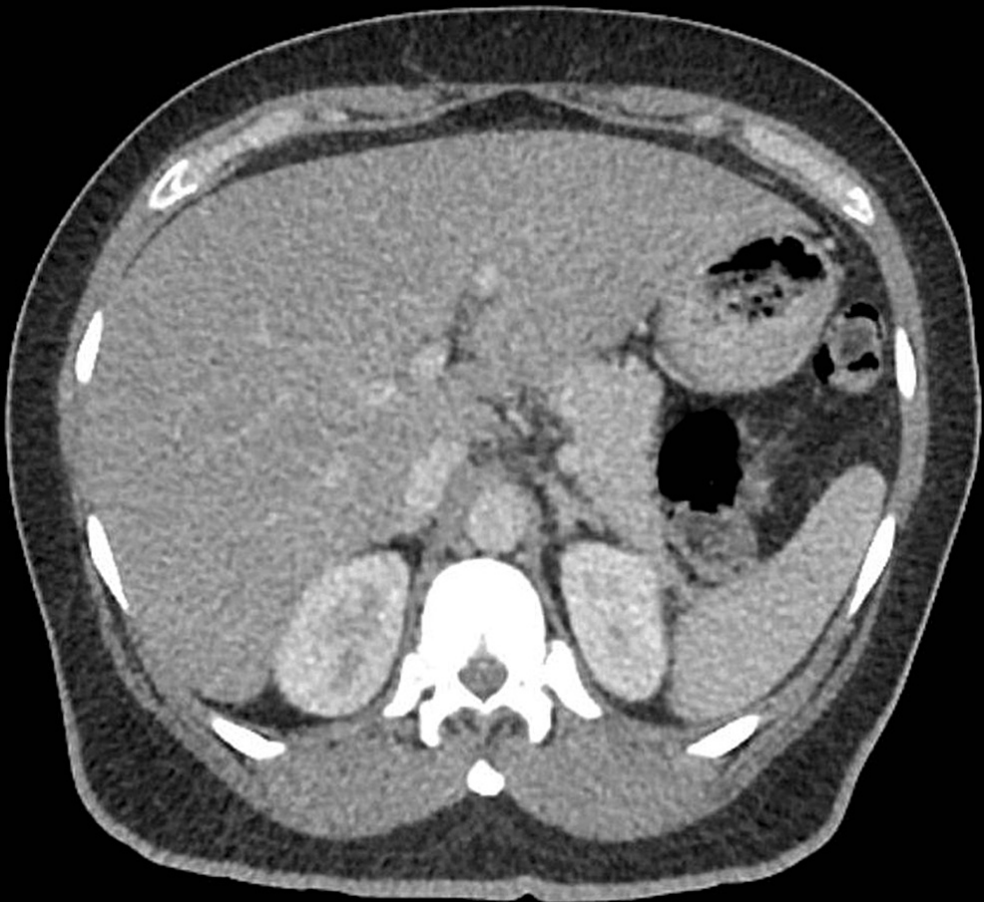
Abdominal computer tomography (CT) findings: hepatomegaly with diffuse hepatic steatosis.

**Figure 4:**
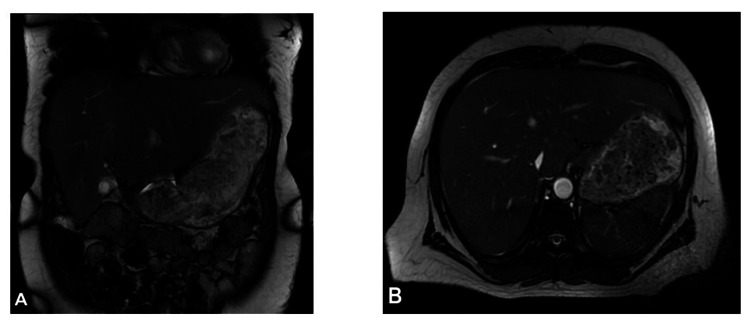
Abdominal magnetic resonance imaging (MRI) findings: (A) There is hepatomegaly with the liver measuring 20.5cm in the craniocaudal dimension (after conservative therapy). (B) A trace amount of perihepatic ascites is present with hepatic and portal veins patent. There is no biliary ductal dilatation, and the gallbladder, spleen, adrenals, and kidneys are within normal limits.

**Figure 5:**
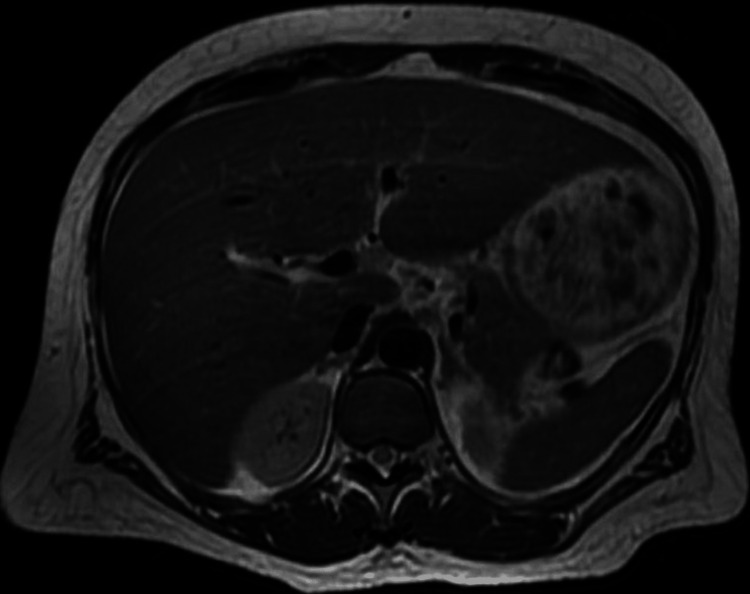
Magnetic resonance cholangiopancreatography (MRCP) findings: The liver is mildly enlarged but homogeneous. No hepatic parenchymal signal loss is demonstrated to suggest hepatic steatosis. The portal veins are patent. The volume of perihepatic ascites is slightly increased. The gallbladder is contracted, but the gallbladder wall does not appear disproportionately thickened, and no gallbladder calculi are identified. There is a small amount of pericholecystic ascites.

**Figure 6:**
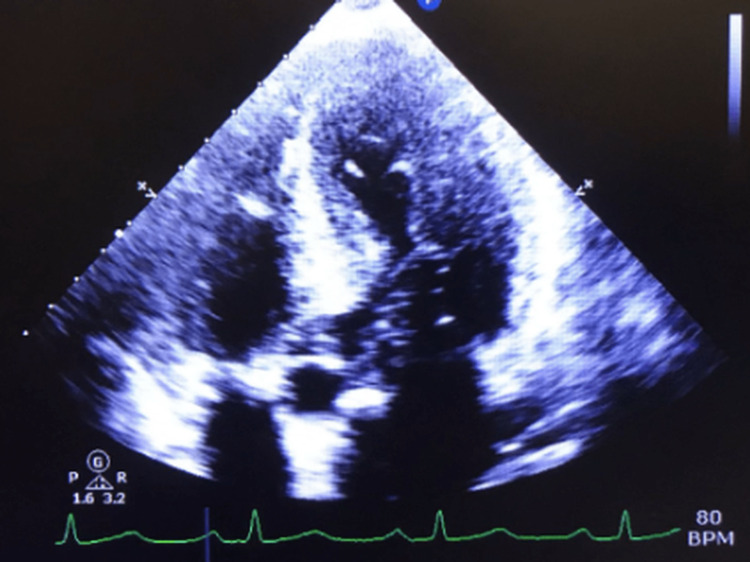
Cardiac transthoracic echocardiogram (TTE) showing infiltrative cardiomyopathy without decreased ejection fraction.

Abdominal ultrasound (Figure 2), computed tomography (CT) (Figure 3), and magnetic resonance imaging (MRI) (Figures 4A, 4B) revealed HM of 20.5 cm with surface nodularity and diffuse steatosis. Serum light chains revealed elevated kappa (κ) 247.8mg/dl and lambda (λ) 16.2mg/dL, SPEP without M spike, and a 24-hour urine κ/λ ratio of 8.64mg/dL (Table 2). Magnetic resonance cholangiopancreatography (MRCP) (Figure 5) confirmed HM with perihepatic ascites without ductal dilation. A transthoracic echocardiogram (TTE) showed infiltrative cardiomyopathy without decreased ejection fraction (Figure 6), and 24-hour urine studies revealed nephrotic syndrome (Table 2).

 

**Table 2:** Imaging, biopsy, and urinalysis results

Imaging	Results
Abdominal Ultrasound (US)	Hepatomegaly measuring 20.68cm and subtle hepatic surface nodularity suggestive of cirrhosis with a moderate volume of ascites (prior to conservative therapy).
Computed Tomography (CT) Scan	Hepatomegaly with diffuse hepatic steatosis.
Magnetic Resonance Imaging (MRI)	Hepatomegaly of 20.5 cm with surface nodularity and diffuse steatosis. no biliary ductal dilatation, hepatic and portal veins are patent, and the gallbladder is unremarkable. A trace amount of perihepatic ascites present (after conservative therapy).
Magnetic resonance cholangiopancreatography (MRCP)	Hepatomegaly with perihepatic ascites without ductal dilation.
Liver Biopsy	Diffusely expanded sinusoids by amorphous eosinophilic deposits were seen on the hematotoxylin and eosin (H&E) stain. Patchy apple-green birefringence was appreciated with congo red stain under polarized and nonpolarized light filters. The trichome stain demonstrated an absence of fibrosis. In addition, amyloid light-chain (AL) kappa amyloidosis was distinguished from AL lambda amyloidosis and discerned from amyloid A (AA) protein by immunohistochemical (IMH) stain.
Computed Tomographic Angiography (CTA)	A significant volume of complex ascites present with a hemorrhagic component.
Cardiac Transthoracic Echocardiography (TTE)	Infiltrative cardiomyopathy without decreased ejection fraction.
24-hour urine studies	3.5 grams of protein
Bone marrow biopsy	10% kappa-restricted plasma cells.

**Figure 7:**
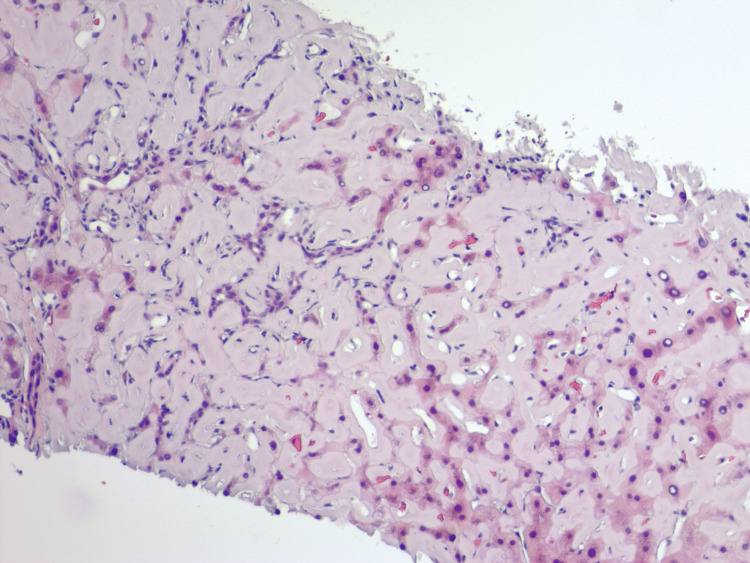
Liver biopsy demonstrating diffusely expanded sinusoids by amorphous eosinophilic deposits infiltrating the portal vessel walls on hematoxylin and eosin stain.

**Figure 8:**
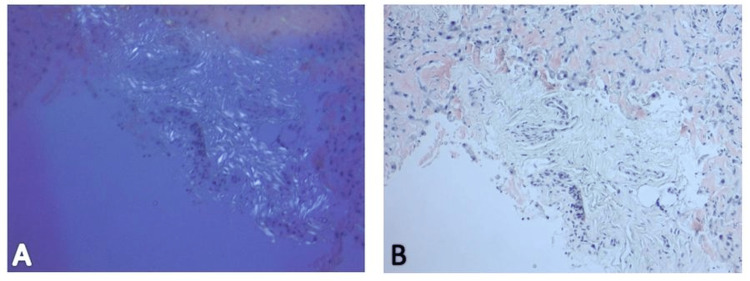
Liver biopsy demonstrating amyloidosis deposition: (A) Patchy apple-green birefringence by congo red stain with a polarizing light filter. (B) Patchy apple-green birefringence by congo red stain with a nonpolarizing light filter.

**Figure 9:**
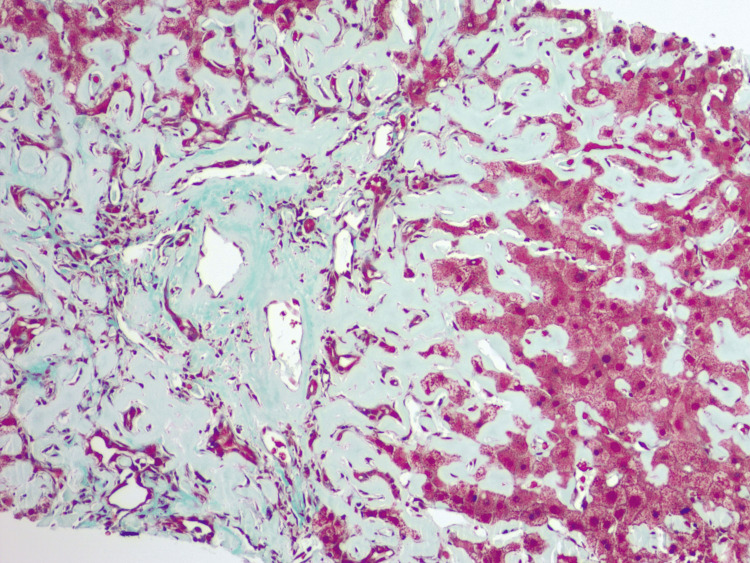
Liver biopsy demonstrating liver parenchyma negative for fibrosis on trichrome stain.

**Figure 10:**
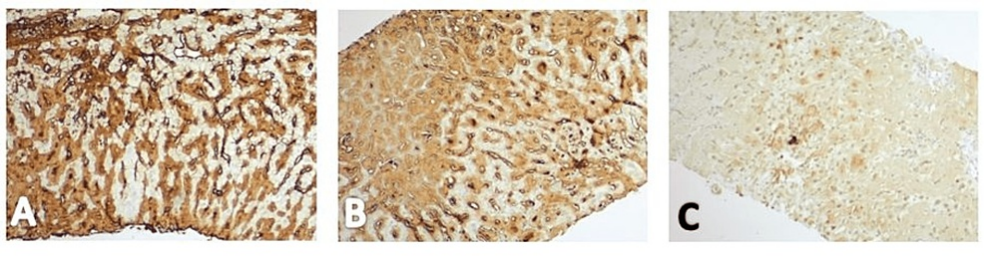
Liver biopsy demonstrating staining of amyloid light-chain (kappa and lambda) and amyloid A protein deposition on immunohistochemical staining. (A) Positive staining of kappa-type amyloid light-chain deposition. (B) Positive staining of lambda-type amyloid light-chain deposition. (C) Positive staining of amyloid A protein deposition.

Due to non-relenting elevation in transaminases, further investigation required biopsies. Liver biopsy revealed diffusely expanded sinusoids by amorphous eosinophilic deposits on hematoxylin and eosin stain (Figure 7) with patchy apple-green birefringence with congo red stain under polarized (Figure 8A) and nonpolarized (Figure 8B) light filters, and negative for fibrosis (Figure 9), as well as positive for AL (Figures 10A, 10B) and AA proteins (Figure 10C) on immunohistochemical (IMH) stain (Table 2). The biopsies showed no evidence of steatosis reported in the imaging, likely due to local amyloid deposition obscuring the view.

 

**Figure 11:**
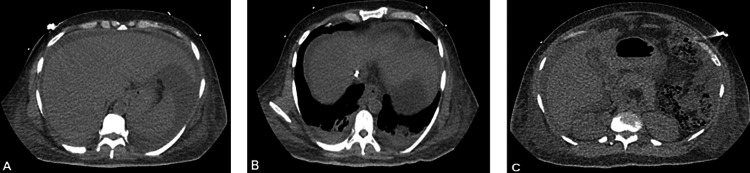
Abdominal computer tomographic angiography (CTA) after liver biopsy findings: (A) Status post-ultrasound-guided core liver biopsy showing the development of new large volume complex attenuating perihepatic ascites fluid tracking along the paracolic gutters into the lower pelvis. This finding may represent blood products. (B) There is an enhancing focal area not seen elsewhere in the liver with a surrounding slight lucency at the periphery of the liver anteriorly suspicious for a site of active extravasation of contrast, likely active bleeding. (C) Diffuse hepatic steatosis with extensive volume complex ascites with centralization of the bowel loops and hematocrit levels in the right paracolic gutter and lower pelvis indicate hemorrhagic ascites or ascites with a hemorrhagic component.

However, following the biopsy, a computed tomographic angiography (Figure 11) showed a significant volume of complex ascites with a hemorrhagic component, suggesting worsening liver failure. Bone marrow biopsy revealed 10% κ-restricted plasma cells. Further staining studies of liver biopsies showed AL-κ amyloidosis (Figure 10A) and AL-λamyloidosis (Figure 10B), as well as AA protein on IMH staining (Figure 10C). The patient left the hospital against medical advice but returned shortly after experiencing a syncopal episode. When re-evaluated, the patient had dyspnea with increasing abdominal distension and severe abdominal pain. Despite abdominal paracentesis to alleviate dyspnea, the patient became hypotensive, requiring vasopressors due to subsequent hemorrhagic shock. After a maximal medical intervention, including intubation, blood products, immunosuppressives, and vasopressors, while in critical care, the patient expired shortly after.

## Discussion

Determining the type of amyloid and underlying etiology is essential to guide the appropriate therapy. AL amyloidosis should be suspected in patients with non-diabetic nephrotic syndrome, non-ischemic cardiomyopathy with hypertrophy on echocardiography, HM, elevated alkaline phosphatase without imaging abnormalities, chronic inflammatory demyelinating polyneuropathy with a monoclonal protein, or the presence of monoclonal gammopathy in a patient with unexplained fatigue, edema, weight loss, or paresthesias [4,5]. AA amyloidosis consists of proteins derived from serum amyloid-associated protein, an APR released during inflammatory states [3,5,6]. AA amyloidosis should be suspected in viral infections, and chronic inflammatory conditions including autoimmune disorders, inflammatory bowel diseases, chronic osteomyelitis, Familial Mediterranean Fever, and neoplasias [3,5,6].

Amyloidosis should be suspected when there is the involvement of various organs where characteristically, amyloid deposits interfere with their function [2,3]. Some examples include proteinuria, HM, elevated alkaline phosphatase, restrictive cardiomyopathy, neuropathy, unexplained edema, carpal tunnel syndrome, unexplained facial or neck purpura, or macroglossia [2,3].

Ultimately, the diagnosis of amyloidosis is established by: (1) tissue biopsy with positive staining of amyloid by congo red stain leading to apple-green birefringence under polarized or non-polarized light filters, (2) Thioflavin-T stain producing an intense yellow-green fluorescence, or [3] by the presence of amyloid fibrils on electron microscopy [3,7,8]. Furthermore, hematoxylin-and-eosin-stained biopsy sections will demonstrate amyloid as it appears as a pink, amorphous, waxy substance with cracking artifacts [8].

Hepatocytes mainly produce serum AA (SAA) in response to inflammatory cytokines such as tumor necrosis factor-α (TNF-α), interleukin-1β (IL-1β), and interleukin-6 (IL-6) [3,7,8]. Therefore, increased synthesis in the liver triggered by several stimuli, including TNF-α, IL-1β, IL-6, and interferon-ɣ (IFN-ɣ), can rapidly induce up to 1,000-fold production of SAA within the first 24-48 hrs of acute phase response in a synergistic manner [7]. This increase in cytokines may explain the rapid induction of acute-phase reactants during the initial phase of COVID-19 infection [7].

A systematic review and meta-analysis that studied 44 articles with 7,865 patients showed a significant decrease in lymphocytes, monocyte, CD4+ T cells, CD8+ T cells, CD3 cells, CD19 cells, and natural killer cells with an increase in the white blood cells, neutrophils, neutrophil to lymphocyte ratio, C-reactive protein, erythrocyte sedimentation rate, ferritin, procalcitonin, and serum amyloid A, interleukins-2, 4, 6, 8, and 10, TNF-α, and IFN-ɣ in patients infected with COVID-19 [7]. In addition, findings concluded that SAA levels shared a significantly close association with COVID-19 severity [7].

Amyloid accumulates in various body parts, including the gastrointestinal tract and liver, with complications resulting in abdominal pain, dysmotility, diarrhea, gastrointestinal bleeding, weight loss, mild HM, and portal hypertension [5,7]. In 2003, the clinical features and natural history were studied in 98 patients with primary hepatic amyloidosis, describing weight loss at 72%, with a median of 10.4 kg (~22.9 lbs). Other symptoms included fatigue (60%), abdominal pain (53%), edema (26%), anorexia (26%), early satiety (19%), nausea (15%), and dysgeusia (10%) [1]. The most common physical exam finding was HM in 81% of patients with a median extension of 8cm below the right costal margin. Other findings included ascites (42%), purpura (15%), splenomegaly (10%), spider angiomata (7%), and macroglossia (1%) [1]. Liver biopsy reported amyloid deposition in 79% of patients and showed solely sinusoidal (66%), solely vascular (13%), and both vascular and sinusoidal (21%) depositions [1].

Systemic amyloidosis with hepatic involvement has been reported in up to 90% of patients with AL amyloid and 60% of patients with AA amyloid [5]. In autopsy series studies, 56-95% of patients with systemic amyloidosis had hepatic involvement [5]. It was present in 62%-90% with AL amyloidosis, 22%-43% with multiple myeloma-associated amyloidoses, and 59%-60% with AA amyloidosis [5].

**Figure 12:**
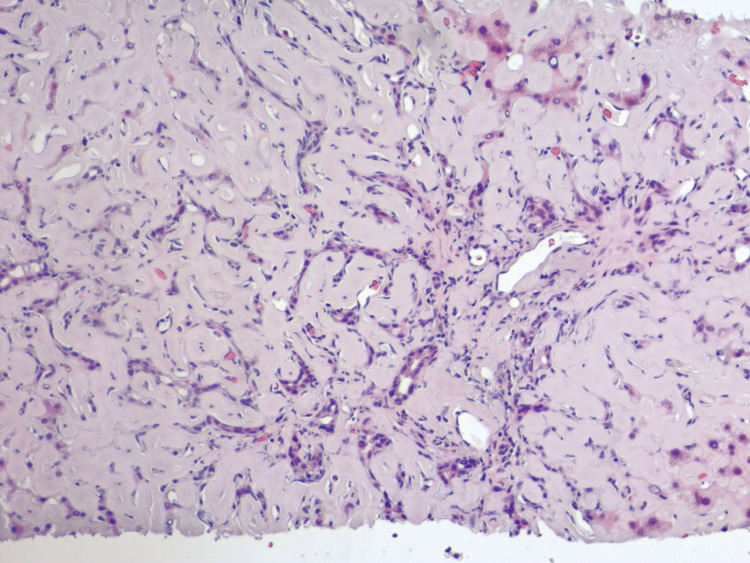
Liver biopsy demonstrating prominent bile ductular proliferation and pressure atrophy of hepatocytes forming cord-like islands on hematoxylin and eosin stain.

**Figure 13:**
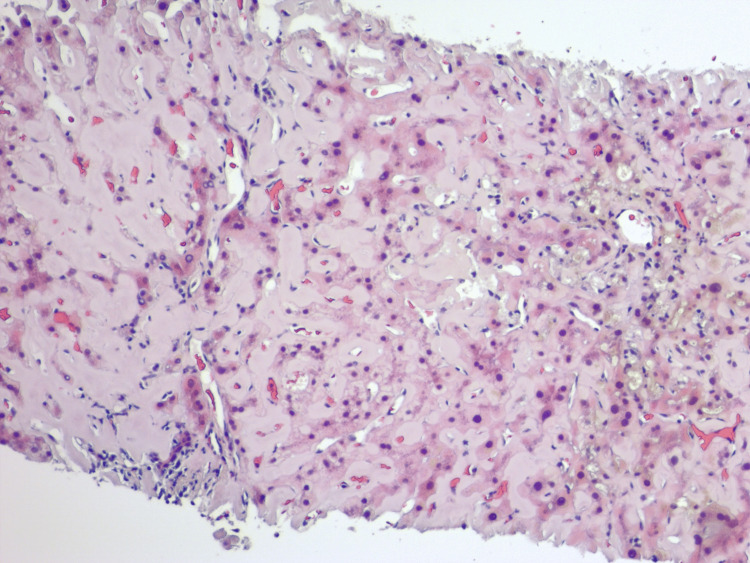
Liver biopsy demonstrating focal canalicular cholestasis and interference with the passage of bile resulting from pressure atrophy of hepatocytes on hematoxylin and eosin stain.

In cases of amyloidosis and concurrent hepatic involvement, amyloid deposits usually begin in the periportal space of Disse, followed by atrophy of the hepatocytes due to compression by amyloid fibrils. However, the disease may occasionally be predominantly centrilobular [4]. When amyloid blocks the sinusoids, portal hypertension develops over time [3,5]. On some occasions, amyloid infiltrates the portal blood vessel walls with globular inclusions within stromal cells, Kupffer cells, and hepatocytes [3]. In severe disease, amyloid can replace parenchyma, leading to pressure atrophy of the hepatocytes, thus forming cord-like islands and interfering with the passage of bile (Figure 12), resulting in the proliferation of small bile ducts, centrilobular canalicular cholestasis (Figure 13), and bile thrombi [3]. Currently, there is no evidence suggesting different patterns of deposition between AL and AA amyloidosis [3,8].

The prognosis of hepatic amyloidosis can vary significantly from patient to patient. Some factors that may play a role may include but are not limited to age, gender, co-existing heart disease, hypertension, diabetes, obesity, and many other chronic diseases. In 1988, Gertz and Kyle described the natural history of primary hepatic amyloidosis in 80 patients [2]. The overall median survival was nine months, 13% at five years, and 1% at 10 years [2]. The study of primary hepatic amyloidosis in 98 patients in 2003 found that the overall median survival was 8.5 months, 16.9% at five years, and 6.6% at 10 years [1]. Our patient with systemic amyloidosis and predominant hepatic involvement survived four months after being admitted and two months after the diagnosis was established. Treatment of primary hepatic amyloidosis and systemic amyloidosis aims to suppress the acute phase responses with anti-inflammatory and immunosuppressive medications with high response and relapse rates of up to 63% and 38.5%, respectively, in heavily treated patients [9,10]. Ultimately the only definitive treatment is liver transplantation.

Although HM is the most common finding, it does not always correlate with the amount of amyloid deposited [3]. It is as common in those primarily with vascular involvement as with parenchymal infiltration [3]. Liver function abnormalities do not correlate with the extent of hepatic infiltration, with primary vs. secondary disease, or with vascular vs. parenchymal disease [3]. Stigmata of chronic liver disease and portal hypertension are also rare [3,4]. Hyperbilirubinemia, found in only 5% of patients, is associated with a poor prognosis, as seen in this patient [3]. Ascites, which are seen more frequently with systemic amyloidosis, are more associated with renal or cardiac dysfunction secondary to AL depositions and hypoalbuminemia and less likely related to hepatic amyloidosis [4,6]. Jaundice resulting from amyloid-induced cholestatic liver disease is extremely rare [3,6]. Splenomegaly is mainly associated with HM and hyposplenism from amyloid infiltration, as indicated by the presence of Howell-Jolly bodies, but this does not correlate with splenomegaly [3].

In 2003, Park et al. described liver biopsy pathology reports of amyloid deposition patterns in 79% of studied patients, some of which expressed either solely sinusoidal (66%), solely vascular (13%), or both vascular and sinusoidal (21%) depositions [1]. Other extra-hepatic biopsies positive for amyloid include fat aspirate biopsy (80%), bone marrow biopsy (82%), and rectal biopsy (65%) [1].

In extra-hepatic involvement, gastrointestinal amyloidosis should be suspected in patients with diarrhea, weight loss, or GI bleeding, as well as disorders that are known to be associated with amyloidosis, including plasma cell dyscrasias, chronic inflammatory diseases, and chronic renal failure on hemodialysis [8]. In contrast, cardiac and renal systemic amyloidosis involvement is much more common than that of hepatic or GI [2,4,5].

COVID-19 and amyloidosis

COVID-19 infection can present with mild, moderate, or severe symptoms. An excessive inflammatory response has been associated with high disease severity and mortality [6,8,11]. In April 2021, a systematic review and meta-analysis were conducted on nineteen studies involving 5,617 COVID-19 patients reporting SAA concentrations, COVID-19 severity, and survival status to assess risk stratification [7]. Pooled results showed that SAA concentrations were significantly higher in patients with severe disease and non-survivors (p < 0.001). They concluded that SAA concentrations were significantly associated with higher COVID-19 severity and mortality.

Moreover, the extent of amyloid deposition has been correlated with morbidity and mortality. In another retrospective cohort study performed in Linhai, China, SAA levels were obtained in 95 COVID-19-infected patients on admission and discharge. It was demonstrated that SAA changes were more significant than CRP, lymphocytes, and neutrophils in correlating with COVID-19 severity, thereby further suggesting an essential role for SAA levels as a valuable biomarker for monitoring the clinical course of COVID-19 patients [6]. Furthermore, SAA levels were found to be markedly elevated during the acute phase and significantly decreased as patients improved with treatment.

In another observational study, 118 patients with COVID-19 infection were followed closely during hospital admission, and SAA, C-reactive protein, and procalcitonin were measured and monitored from day one to day fourteen [11]. SAA was found to have an accuracy of 89.1% in predicting acute exacerbations of COVID-19, suggesting a role as an independent reliable predictive factor of COVID-19 severity. Unfortunately, despite the supporting facts mentioned above, SAA levels were not obtained in our patient but were confirmed with liver biopsy reports.

COVID-19 is a systemic viral illness that took the world by surprise and has proven that it can have many different manifestations. The liver is a vital organ that houses some of those diverse clinical manifestations. Since the COVID-19 pandemic began, it has been reported that liver enzymes were elevated in 14% to 53% of patients infected, with AST being more predominant than ALT in viral hepatitis [11]. Patients with COVID-19 may also suffer from prothrombotic complications, leading to significant hepatic injury through portal venous thrombosis. More research is required regarding pre-existing hepatic disease and subsequent infection with COVID-19.

## Conclusions

Systemic amyloidosis with hepatic involvement is a rare disease manifestation. The extent of involvement and degree of worsening clinical picture of this case where we see primary and secondary systemic amyloidosis makes this case extremely rare. Recent studies confirm that COVID-19 patients have high levels of circulating amyloid protein, SAA, as part of the APRs and disease course. In addition to an active or maybe dormant underlying systemic primary amyloidosis with predominant hepatic involvement, in this case, we hypothesize that infection with COVID-19 provoked a compelling immune response that contributed to the activation or accelerated progression of the natural course of systemic amyloidosis. Our patient had several negative COVID-19 polymerase chain reaction (PCR) tests and a positive IgG test, throughout the hospital course. Although false negatives have been previously documented on the COVID-19 antibody tests, it begs one to wonder how COVID-19 infection may accelerate the natural course of systemic amyloidosis. Further evidence and investigation are needed to establish or disregard the correlation between AL and AA amyloid protein deposits in amyloidosis and how they may act synergistically to exacerbate an already complicated clinical course.
